# Cytokine profiles in the aqueous humor of uveal melanoma: implications for tumor progression and radiation response

**DOI:** 10.3389/fmed.2025.1658817

**Published:** 2025-09-19

**Authors:** Jie Ding, Han Yue, Binbin Xu, Lianyao Shi, Jie Guo, Jiang Qian

**Affiliations:** ^1^Department of Ophthalmology, Eye and ENT Hospital of Fudan University, Shanghai, China; ^2^Shanghai Key Laboratory of Visual Impairment and Restoration, Shanghai, China

**Keywords:** uveal melanoma, inflammatory cytokines, tumor microenvironment, radiotherapy, eye, aqueous humor

## Abstract

**Purpose:**

This study aimed to investigate the cytokine profile in the aqueous humor of uveal melanoma (UM) and explore the role of cytokines in tumor progression and radiation response.

**Methods:**

Aqueous humor samples were collected from patients with UM who had undergone enucleation or radiotherapy. Cytokine levels in these samples were measured using the Human 48-Plex Luminex assay, and statistical analyses were performed to evaluate the correlations with clinicopathological parameters.

**Results:**

Thirty-six patients with UM were enrolled in this study: 17 in the enucleation group and 19 in the radiotherapy group. Significant differences in cytokine levels were observed between the two groups. The enucleation group exhibited higher levels of basic fibroblast growth factor (basic-FGF), interleukin-2 (IL-2), interleukin-2 receptor alpha (IL-2Rα), interleukin-12 subunit p40 (IL-12(P40)), macrophage colony-stimulating factor (M-CSF), macrophage migration inhibitory factor (MIF) than the radiotherapy group. Correlation analysis revealed significant associations between several cytokines and clinicopathological features, including tumor height, diameter, and treatment strategy. Tumor height was correlated with cytokines such as interleukin-8 (IL-8), M-CSF, and stem cell growth factor-beta (SCGF-β), while tumor diameter showed negative correlations with beta-nerve growth factor (β-NGF) and tumor necrosis factor-beta (TNF-β). Cytokines, including basic-FGF, IL-2, and MIF, were positively associated with radiation complications, while cytokines such as β-NGF, interleukin-12 subunit p70 (IL-12(P70)), and vascular endothelial growth factor (VEGF) exhibited negative correlations with post-radiation duration. Interleukin-5 (IL-5) was the only cytokine linked to subretinal fluid, and multiple cytokines, such as M-CSF and MIF, were correlated with the tumor cell type. Multiple regression analysis confirmed that these cytokines, including interferon-gamma (IFN-γ), interleukin-1 receptor antagonist (IL-1ra) and VEGF, were significantly associated with tumor characteristics and treatment response.

**Conclusion:**

These findings suggest the potential role of cytokines in the tumor microenvironment of UM. The analysis points to these cytokines as possible biomarkers for tumor progression and treatment response. Further exploration of cytokines in the aqueous humor may enhance our understanding of UM and provide insights for managing post-treatment complications.

## 1 Introduction

Uveal melanoma (UM) is the most common primary malignancy in adults, which originates from pigmented cells of the uvea (1). The treatment options for UM include enucleation, radiotherapy, local excision, and laser therapy. Despite the availability of various local treatments, nearly half of the patients still develop systemic metastasis, which is typically associated with a poor prognosis. Currently, recognized prognostic factors for UM include tumor size, location, cell type, and genetic mutations (2). However, identifying high-risk patients early remains challenging even with these prognostic tools. In this context, cytokine analysis of the aqueous humor through liquid biopsy is promising, offering a minimally invasive technique for real-time and continuous molecular evaluation of the primary tumor.

Recent research has suggested that pathways related to angiogenesis, immunity, and inflammation contribute to the progression and dissemination of UM (3, 4). Cytokines, chemokines, and growth factors, which activate and regulate these processes, are increasingly recognized for their importance in tumor diagnostics and treatment. Analyzing these factors in the aqueous humor can provide critical insights into intraocular inflammation, tumor microenvironment, and responses to treatment.

In this study, we examined cytokine levels in the aqueous humor of patients with UM after enucleation and radiotherapy and investigated their associations with clinical parameters. This study aimed to offer new insights and identify potential biomarkers for UM progression and treatment response.

## 2 Materials and methods

### 2.1 Ethics approval

The study adhered to the principles of the Declaration of Helsinki. All patients provided written informed consent, and institutional ethics committee approval was obtained (2020044-1).

### 2.2 Patients and samples

Aqueous humor samples from 36 patients with UM were obtained from the Eye and ENT Hospital of Fudan University between 2022 and 2023. The exclusion criteria included concomitant ocular diseases, severe systemic diseases such as diabetes, and previous intraocular surgery.

Each enrolled patient underwent a complete ophthalmological examination, including slit-lamp biomicroscopy, ophthalmoscopy, and B-scan ultrasonography. An experienced ocular oncologist established the clinical diagnosis of UM. Tumor–node–metastasis (TNM) staging was performed according to the 8th edition of the American Joint Committee on Cancer (AJCC) classification system (5).

Patients were considered eligible for radiotherapy if they had moderate-sized tumors (stage T2 or T3) without significant complications, such as cataract, total retinal detachment, or dense vitreous hemorrhage, and had no evidence of metastasis. A preference for eye-preserving treatment and sufficient financial resources to afford radiotherapy was also required. Enucleation surgery is recommended for patients with larger tumors (stage T3 or T4) or those with severe complications, including neovascular glaucoma or markedly decreased visual acuity.

Patients in the enucleation group underwent surgery on the affected eye without prior treatment, whereas those in the radiotherapy group received heavy-ion radiotherapy with a total dose of 45 GyE. Demographic and clinical data, including age, sex, laterality, and duration post-radiation, were collected, and tumor features, such as basal tumor diameter, tumor height, and ciliary body involvement, were recorded.

Aqueous humor samples were collected after enucleation in the enucleation group and before intravitreal injection in the radiotherapy group following standard sterilization procedures. A paracentesis was performed at the temporal limbus under aseptic conditions. Approximately 100–150 μL of aqueous humor was carefully aspirated using a 1-mL syringe. Aspiration was performed with caution to avoid contamination with blood or iris tissue. The undiluted samples were immediately transferred into sterile microtubes and stored at −80 °C until tested.

### 2.3 Aqueous humor analysis

Luminex liquid suspension array analysis was performed by Wayen Biotechnologies (Shanghai, China). The Human 48-Plex Luminex assay was performed according to the manufacturer’s instructions. Forty-eight cytokines were selected to comprehensively represent major classes of cytokines, including interleukins, interferons, tumor necrosis factors, chemokines, and growth factors (6, 7). These cytokines have been widely studied and validated in biological processes such as inflammation, immune regulation, angiogenesis, and modulation of the tumor microenvironment (8–10). The cytokines measured included cutaneous T-cell attracting chemokine (CTACK), Eotaxin, basic fibroblast growth factor (basic-FGF), granulocyte colony-stimulating factor (G-CSF), granulocyte-macrophage colony-stimulating factor (GM-CSF), growth-regulated oncogene-alpha (GRO-α), hepatocyte growth factor (HGF), interferon-alpha 2 (IFN-α2), interferon-gamma (IFN-γ), interleukin-1 alpha (IL-1α), interleukin-1 beta (IL-1β), interleukin-1 receptor antagonist (IL-1ra), interleukin-2 (IL-2), interleukin-2 receptor alpha (IL-2Rα), interleukin-3 (IL-3), interleukin-4 (IL-4), interleukin-5 (IL-5), interleukin-6 (IL-6), interleukin-7 (IL-7), interleukin-8 (IL-8), interleukin-9 (IL-9), interleukin-10 (IL-10), interleukin-12 subunit P40 (IL-12(P40)), interleukin-12 subunit p70 (IL-12(P70)), interleukin-13 (IL-13), interleukin-15 (IL-15), interleukin-16 (IL-16), interleukin-17 (IL-17), interleukin-18 (IL-18), interferon gamma-induced protein 10 (IP-10), leukemia inhibitory factor (LIF), monocyte chemoattractant protein-1 (MCP-1), monocyte chemoattractant protein-3 (MCP-3), macrophage colony-stimulating factor (M-CSF), macrophage migration inhibitory factor (MIF), monokine induced by gamma interferon (MIG), macrophage inflammatory protein-1 alpha (MIP-1α), macrophage inflammatory protein-1 beta (MIP-1β), beta-nerve growth factor (β-NGF), platelet-derived growth factor-BB (PDGF-BB), regulated on activation, normal T cell expressed and secreted (RANTES), stem cell factor (SCF), stem cell growth factor-beta (SCGF-β), stromal cell-derived factor-1 alpha (SDF-1α), tumor necrosis factor-alpha (TNF-α), tumor necrosis factor-beta (TNF-β), TNF-related apoptosis-inducing ligand (TRAIL), and vascular endothelial growth factor (VEGF).

### 2.4 Statistical analysis

Statistical analyses were performed, and graphics were generated using R 4.3.3 and GraphPad Prism 8 software. Statistical differences between the two groups were assessed using an unpaired two-tailed Student’s *t*-test. Pearson’s correlation analysis and multiple linear regression examined the relationships between the cytokine levels and clinical features. Statistical significance was set at *p* < 0.05.

## 3 Results

### 3.1 Clinical characteristics of patients with UM

Thirty-six patients with UM were enrolled in this study and grouped into enucleation (*n* = 17) and radiotherapy (*n* = 19) groups based on the treatment modality. The average age of patients in the enucleation group was 51.59 ± 16.21 years compared to 43.63 ± 12.82 years in the radiotherapy group. The sex distribution was similar across the groups, with males constituting 47.06% (*n* = 8) of the enucleation group and 57.89% (*n* = 11) of the radiotherapy group. Females accounted for 52.94% (*n* = 9) in the enucleation group and 42.11% (*n* = 8) in the radiotherapy group. Regarding the affected eye, 58.82% (*n* = 10) of patients in the enucleation group had right eye (OD) involvement compared to 36.84% (*n* = 7) in the radiotherapy group. Left eye (OS) involvement was reported in 41.18% (*n* = 7) of patients in the enucleation group and 63.16% (*n* = 12) of patients in the radiotherapy group. The basal tumor diameter was larger in the enucleation group, with a mean of 13.04 ± 3.30 mm, in contrast to 10.50 ± 3.09 mm in the radiotherapy group. Tumor height also differed, with a mean of 9.94 ± 5.56 mm in the enucleation group and 6.77 ± 4.42 mm in the radiotherapy group. Ciliary body involvement was reported in 47.06% (*n* = 8) of patients in the enucleation group. Histopathological analysis of the enucleation group revealed that 41.18% (*n* = 7) of patients had the spindle cell type, 35.29% (*n* = 6) had the mixed cell type, and 23.53% (*n* = 4) were undetermined. The presence of subretinal fluid was reported in 64.71% (*n* = 11) of patients in the enucleation group and 68.42% (*n* = 13) of the radiotherapy group. For the radiotherapy group, the average duration post-radiation was 7.61 ± 7.47 months, and 63.16% (*n* = 12) of patients in the radiotherapy group developed radiation-related complications. The most common complications included radiation retinopathy, retinal detachment, and cataract formation. The clinical and histopathological features of patients with UM are summarized in Table 1.

**Table 1:** Clinical and histopathological characteristics of patients with UM.

Variable	Patients, *n* (%)	
	Enucleation group, *n* = 17	Radiotherapy group, *n* = 19
Age (year, Mean ± SD)	51.59 ± 16.21	43.63 ± 12.82
Sex		
Male	8 (47.06%)	11 (57.89%)
Female	9 (52.94%)	8 (42.11%)
Eye		
OD	10 (58.82%)	7 (36.84%)
OS	7 (41.18%)	12 (63.16%)
Basal tumor diameter (mm)	13.04 ± 3.30	10.50 ± 3.09
Tumor height (mm)	9.94 ± 5.56	6.77 ± 4.42
Ciliary body involvement		
Yes	8 (47.06%)	N/A
No	9 (52.94%)	N/A
Histopathological cell type		
Spindle	7 (41.18%)	N/A
Mixed	6 (35.29%)	N/A
Undetermined	4 (23.53%)	N/A
Subretinal fluid		
Yes	11 (64.71%)	13 (68.42%)
No	6 (35.29%)	6 (31.58%)
Duration post-radiation (month, Mean ± SD)	N/A	7.61 ± 7.47
Radiation complications		
Yes	N/A	12 (63.16%)
No	N/A	7 (36.84%)

### 3.2 Comparison of cytokine levels in UM

A comprehensive cytokine profile analysis was performed on aqueous humor samples from patients with UM. The median and mean cytokine levels that differed significantly between the two groups are listed in Table 2. Notably, the enucleation group exhibited significantly higher levels of basic-FGF with a concentration of 35.69 ± 36.15 pg/mL compared to 12.96 ± 7.13 pg/mL in the radiotherapy group (*p* = 0.021). IL-2 levels were also higher in the enucleation group, at 4.78 ± 1.21 pg/mL, compared to 3.15 ± 1.35 pg/mL in the radiotherapy group (*p* = 0.001). Similarly, increased IL-2Rα level of 38.76 ± 9.92 pg/mL was found in the enucleation group compared to 23.39 ± 13.80 pg/mL in the radiotherapy group (*p* = 0.001). The enucleation group also exhibited a significantly higher level of IL-12, with 1.20 ± 0.61 pg/mL versus 0.62 ± 0.49 pg/mL of the radiotherapy group (*p* = 0.004). In addition, M-CSF and MIF levels were significantly elevated in the enucleation group with concentrations of 14.76 ± 13.61 pg/mL (*p* = 0.046) and 402.21 ± 314.24 pg/mL (*p* = 0.001) compared to 7.02 ± 7.03 pg/mL and 99.39 ± 40.02 pg/mL in the radiotherapy group, respectively.

**Table 2:** Cytokine concentrations (pg/mL) (Mean ± SD) in the aqueous humor that significantly differ between the enucleation and radiotherapy groups in patients with UM.

Cytokines	Enucleation group		Radiotherapy group		*p-*value
	Median	Mean ± SD	Median	Mean ± SD	
Basic-FGF	19.17	35.69 ± 36.15	11.71	12.96 ± 7.13	*0.021*
IL-2	4.83	4.78 ± 1.21	3.19	3.15 ± 1.35	*0.001*
IL-2Rα	35.71	38.76 ± 9.92	17.66	23.39 ± 13.80	*0.001*
IL-12(P40)	1.49	1.20 ± 0.61	0.57	0.62 ± 0.49	*0.004*
M-CSF	11.41	14.76 ± 13.61	3.62	7.02 ± 7.03	*0.046*
MIF	267.08	402.21 ± 314.24	96.59	99.39 ± 40.02	*0.001*

*P* values are shown in italics to denote statistical significance (*p* < 0.05).

### 3.3 Correlation analysis

**Figure 1:**
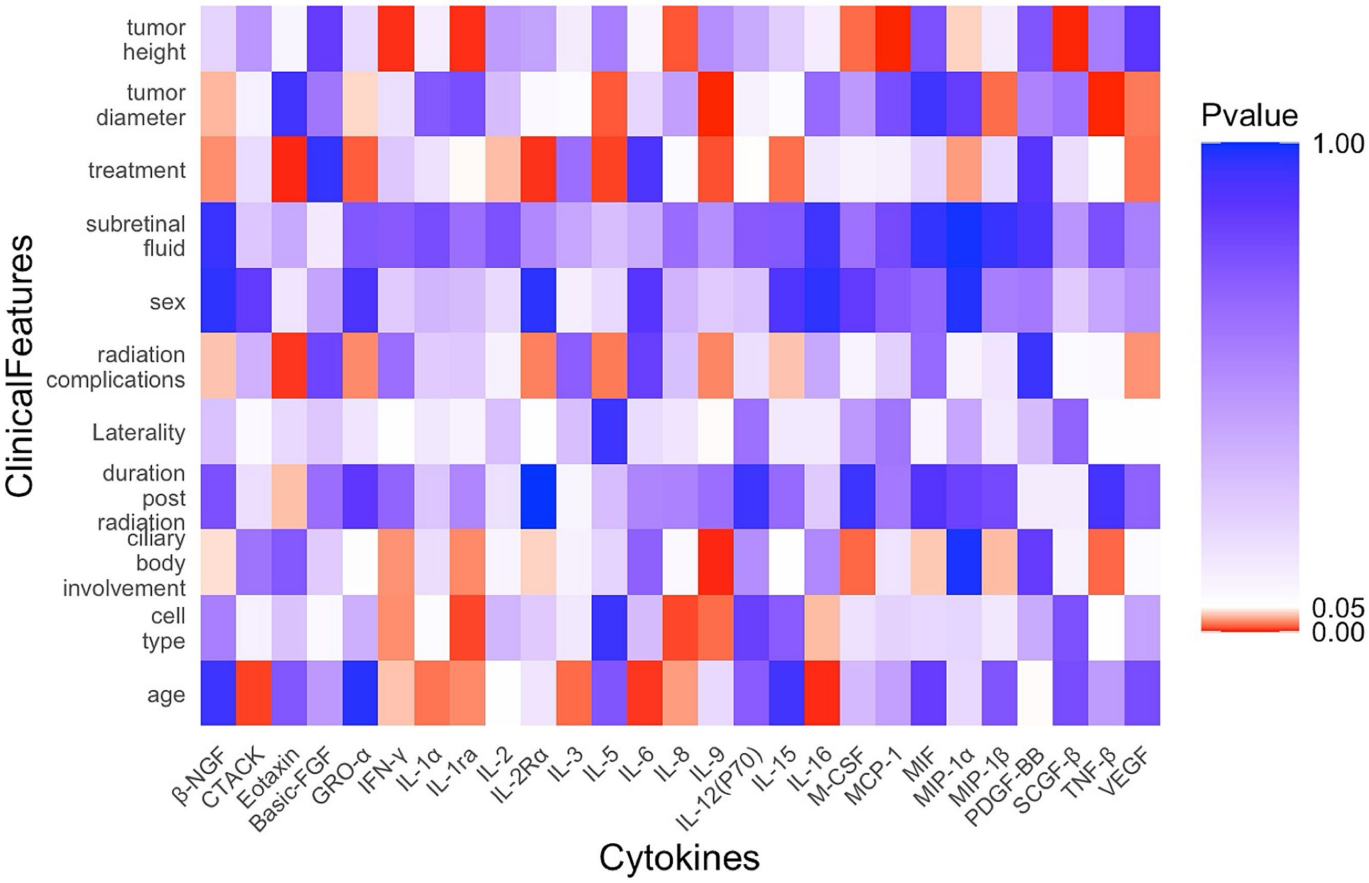
Correlation analysis between cytokine levels and clinical features in patients with UM.

A correlation analysis was conducted to examine the relationships between specific cytokine levels and the clinical features of patients with UM. As shown in Figure 1, a significant correlation was found between tumor height and several cytokines, including β-NGF, CTACK, Eotaxin, IFN-γ, IL-16, IL-1α, IL-1ra, IL-6, IL-8, M-CSF, MCP-1, MIP-1α, and SCGF-β. Tumor diameter showed negative correlations with both β-NGF and TNF-β. The enucleation and radiotherapy treatment strategies were positively correlated with basic-FGF, IL-12(P70), IL-2, IL-2Rα, M-CSF, and MIF, respectively. IL-5 was the only cytokine significantly associated with subretinal fluid. In the radiotherapy group, correlations between cytokine levels and either radiation complications or the duration post-radiation were evaluated separately. Cytokines such as basic-FGF, IL-2, MIF, and SCGF-β showed positive correlations with radiation complications. However, negative correlations were found between the duration post-radiation and several cytokines, including β-NGF, GRO-α, IL-12(P70), IL-15, IL-2, IL-2Rα, IL-3, IL-9, M-CSF, MIF, MIP-1β, PDGF-BB, TNF-β, and VEGF. In the enucleation group, the cell type was related to Eotaxin, basic-FGF, M-CSF, and MIF. HGF, IL-12(P40), IL-16, IL-1β, IL-6, IP-10, and MIF were associated with the age of patients with UM. None of the 48 cytokines showed significant correlations with ciliary body involvement, sex, or laterality in patients with UM.

### 3.4 Regression analysis

**Figure 2:**
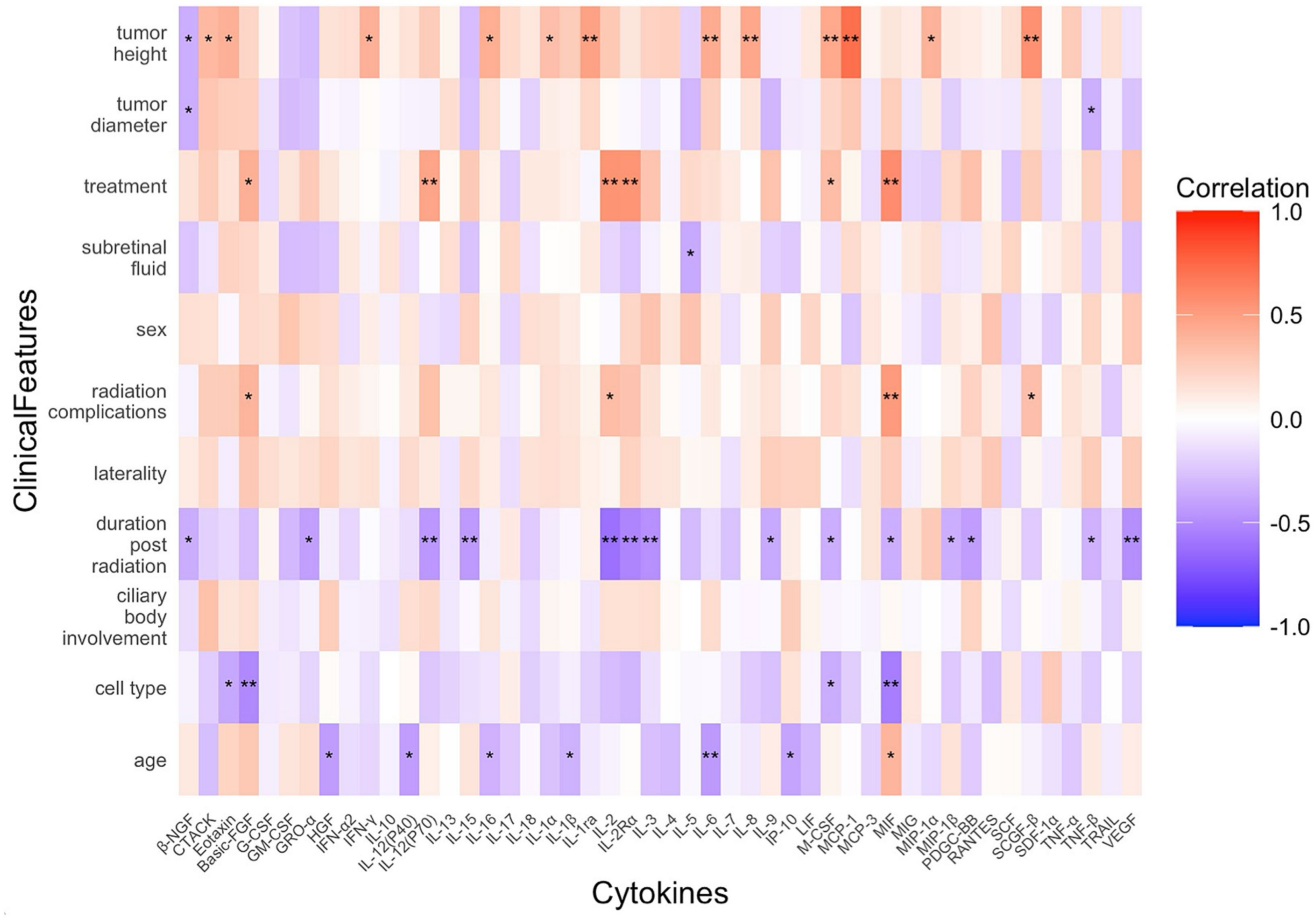
Multiple regression analysis of cytokines significantly associated with clinical features in patients with UM.

Multiple regression analysis was performed for cytokines that showed significant correlations with the clinical features, as illustrated in Figure 2. The analysis revealed that cytokines, including IFN-γ, IL-1ra, IL-8, M-CSF, MCP-1, MIP-1α, and SCGF-β, were associated with tumor height, while β-NGF, GRO-α, IL-5, IL-9, MIP-1β, TNF-β, and VEGF were linked to tumor diameter. β-NGF, Eotaxin, GRO-α, IL-2, IL-2Rα, IL-5, IL-9, IL-15, MIP-1α, and VEGF showed significant associations with treatment strategies. β-NGF, Eotaxin, GRO-α, IL-15, IL-2Rα, IL-5, IL-9, and VEGF were significantly correlated with radiation complications. In addition, Eotaxin was associated with post-radiation duration. Regarding pathological features of UM tumors, β-NGF, IFN-γ, IL-1ra, IL-2Rα, IL-9, M-CSF, MIF, MIP-1β, and TNF-β were associated with ciliary body involvement. In contrast, IFN-γ, IL-16, IL-1ra, IL-8, and IL-9 were related to tumor cell type. In addition, CTACK, IFN-γ, IL-1α, IL-1ra, IL-3, IL-6, IL-8, and IL-16 were associated with the age of patients with UM. No significant correlations were found between cytokines and clinical features, such as subretinal fluid, sex, or laterality.

## 4 Discussion

Despite its relative rarity, UM is the leading primary intraocular tumor in adults worldwide. It originates from the uveal tract, including the iris, ciliary body, and choroid, with most cases occurring in the choroid (11). Although notable advances have been made in treating localized UM, a major challenge remains its high tendency to metastasize, which occurs in approximately 50% of cases and significantly worsens the prognosis (12). Angiogenesis, inflammation, and immunomodulation are critical pathways involved in the pathogenesis of tumors, including UM. These processes contribute to the initial development of UM and influence its progression, metastatic potential, and response to treatment. Recent research has focused on cytokines and their roles in various biological processes (13). This study explored cytokine profiles in aqueous humor samples from patients with UM who underwent enucleation or radiation therapy.

Angiogenesis facilitates tumor growth by supplying nutrients and oxygen through new blood vessels, with cytokines such as VEGF, HGF, basic-FGF, and PDGF-BB being key mediators. Moreover, angiogenesis can contribute to treatment resistance by repairing blood vessels damaged by radiotherapy, potentially facilitating tumor survival following radiation (14). VEGF, basic-FGF, and PDGF have been reported to be highly expressed in the aqueous humor of eyes in patients with UM (15) In patients with UM after Iodine-125 plaque therapy, VEGF levels in the aqueous humor significantly increased with tumor thickness and during the radiation process (16). Our study revealed an increased level of basic-FGF in the enucleation group compared to that in the radiotherapy group, as well as a significant association with radiation complications in the radiotherapy group and with cell type in the enucleation group. Moreover, VEGF, one of the most prominent cytokines involved in angiogenesis, was associated with tumor diameter, radiation complications, and duration post-radiation in our study. PDGF-BB was also associated with the duration following radiation therapy. These findings suggested a possible involvement of angiogenesis in UM tumor progression and response to radiation, underscoring the importance of further investigating the therapeutic potential of anti-angiogenic agents for treating UM and managing post-radiation complications, such as retinal edema.

Elevated levels of inflammatory and chemotactic cytokines were also detected in the aqueous humor of eyes with UM. Usui et al. (17) reported a significant increase in proinflammatory molecules, including IL-8, RANTES, and MCP-1, in the eyes of patients with UM compared to those with benign choroidal nevi. In our study, inflammatory cytokines, including IFN-γ, IL-16, IL-1α, IL-1ra, IL-6, IL-8, IL-9, and TNF-β, were significantly associated with aggressive characteristics of UM.

In addition, IL-2, IL-2Rα, and IL-12(P40) showed increased expression in enucleated eyes with UM and significant correlation with radiation complications and time after radiation. IL-2Rα was also associated with ciliary body involvement in the enucleation group. These inflammatory molecules are involved in the activation of immune cells and the modulation of the inflammatory response (18, 19). Their altered expression may contribute to the aggressive behavior of UM and the enhanced immune microenvironment observed after radiotherapy, indicating a potential role of these cytokines in disease monitoring and as candidates for further investigation. Moreover, our study reported that IL-15, IL-3, IL-5, and IL-9 levels were correlated with the duration or complications of radiation therapy, suggesting that these cytokines merit further study regarding their putative role in managing radiation-related side effects.

Another major focus of the tumor microenvironment is the tumor-associated macrophages (TAMs), representing the dominant population of tumor inflammatory cells. Our study detected multiple cytokines involved in macrophage development, proliferation, and survival, including MCP-1, MCP-3, M-CSF, MIF, MIG, MIP-1α, and MIP-1β. M-CSF is crucial for the differentiation and maintenance of macrophages (20). Increased expression of M-CSF in enucleated eyes with UM and its association with aggressive tumor features, such as tumor height and ciliary body involvement, may be indicative of enhanced macrophage infiltration and activation in the tumor microenvironment, warranting further exploration. Similar correlations were also observed in macrophage chemotactic or modulatory factors, such as MCP-1, MIP-1α, MIP-1β, and MIF. Previous studies have reported that MCP-1 is highly expressed in eyes with UM and is correlated with tumor progression (17, 21, 22). In addition, increased TRAIL and GM-CSF levels were associated with higher HLA class II expression (23). These findings suggest that these cytokines in the tumor microenvironment may serve as biomarkers for aggressive UM and offer insights into potential therapeutic targets.

Other chemokines, such as CTACK, Eotaxin, and GRO-α, were identified and found to correlate with UM aggressiveness and response to radiation. In our study, the neurotrophic factor β-NGF and the hematopoietic growth factor SCGF-β were correlated with tumor size and radiation-related features. However, their role in UM requires further investigation.

To our knowledge, this is the first study to explore the cytokine profiles in patients with UM treated with enucleation and radiotherapy. Although this study highlights the crucial associations between cytokines and UM, several limitations should be acknowledged. The relatively small cohort size may hinder a comprehensive understanding of the complex roles of cytokines in the tumor microenvironment. In addition, the absence of control samples from patients without tumors or with other ocular diseases limits our ability to compare cytokine levels with baseline conditions, making it challenging to fully interpret the alterations observed in patients with UM. Another drawback of this study was the selection bias in cytokine comparisons as the treatment choice depended on tumor size and complications. In addition, the lack of long-term follow-up data, particularly regarding relapses or metastases, limits our ability to assess the long-term impact of cytokine levels on UM progression and their possible clinical utility. Further research involving larger patient cohorts, longitudinal data, and functional studies is needed to validate these findings and to elucidate the precise mechanisms by which cytokines influence UM development and treatment outcomes.

In summary, our findings revealed distinct cytokine profiles associated with aggressive tumor characteristics and responses to radiation. Elevated levels of specific cytokines were linked to tumor progression and radiation-related complications, suggesting their potential as biomarkers for tumor monitoring and therapeutic strategies. These insights enhance our understanding of the molecular mechanisms underlying UM and highlight the need for further research to validate these biomarkers and elucidate their roles in UM.

## Data Availability

The raw data supporting the conclusions of this article will be made available by the authors, without undue reservation.

## References

[1] Singh AD Turell ME Topham AK. Uveal melanoma: trends in incidence, treatment, and survival. Ophthalmology. (2011) 118:1881–5. doi: 10.1016/j.ophtha.2011.01.040, PMID: 21704381

[2] Pašalić D Nikuševa-Martić T Sekovanić A Kaštelan S. Genetic and epigenetic features of uveal melanoma-an overview and clinical implications. Int J Mol Sci. (2023) 24:12807. doi: 10.3390/ijms241612807, PMID: 37628989 PMC10454135

[3] García-Mulero S Alonso MH Del Carpio LP Sanz-Pamplona R Piulats JM. Additive role of immune system infiltration and angiogenesis in uveal melanoma progression. Int J Mol Sci. (2021) 22:2669. doi: 10.3390/ijms22052669, PMID: 33800878 PMC7961481

[4] Castet F Garcia-Mulero S Sanz-Pamplona R Cuellar A Casanovas O Caminal JM. Uveal melanoma, angiogenesis and immunotherapy, is there any Hope? Cancers (Basel). (2019) 11:834. doi: 10.3390/cancers11060834, PMID: 31212986 PMC6627065

[5] Edge SB Edge SB. AJCC cancer staging manual. 8th ed. New York: Springer (2017).

[6] Propper DJ Balkwill FR. Harnessing cytokines and chemokines for cancer therapy. Nat Rev Clin Oncol. (2022) 19:237–53. doi: 10.1038/s41571-021-00588-9, PMID: 34997230

[7] Kureshi CT Dougan SK. Cytokines in cancer. Cancer Cell. (2025) 43:15–35. doi: 10.1016/j.ccell.2024.11.011, PMID: 39672170 PMC11841838

[8] Pradhan R Kundu A Kundu CN. The cytokines in tumor microenvironment: from cancer initiation-elongation-progression to metastatic outgrowth. Crit Rev Oncol Hematol. (2024) 196:104311. doi: 10.1016/j.critrevonc.2024.104311, PMID: 38442808

[9] Xu S Wang Q Ma W. Cytokines and soluble mediators as architects of tumor microenvironment reprogramming in cancer therapy. Cytokine Growth Factor Rev. (2024) 76:12–21. doi: 10.1016/j.cytogfr.2024.02.003, PMID: 38431507

[10] Yi M Li T Niu M Zhang H Wu Y Wu K. Targeting cytokine and chemokine signaling pathways for cancer therapy. Signal Transduct Target Ther. (2024) 9:176. doi: 10.1038/s41392-024-01868-3, PMID: 39034318 PMC11275440

[11] Kaliki S Shields CL. Uveal melanoma: relatively rare but deadly cancer. Eye (Lond). (2017) 31:241–57. doi: 10.1038/eye.2016.275, PMID: 27911450 PMC5306463

[12] Carvajal RD Schwartz GK Tezel T Marr B Francis JH Nathan PD. Metastatic disease from uveal melanoma: treatment options and future prospects. Br J Ophthalmol. (2017) 101:38–44. doi: 10.1136/bjophthalmol-2016-309034, PMID: 27574175 PMC5256122

[13] Chen C Wang Z Ding Y Qin Y. Tumor microenvironment-mediated immune evasion in hepatocellular carcinoma. Front Immunol. (2023) 14:1133308. doi: 10.3389/fimmu.2023.1133308, PMID: 36845131 PMC9950271

[14] Rani V Prabhu A. Combining angiogenesis inhibitors with radiation: advances and challenges in cancer treatment. Curr Pharm Des. (2021) 27:919–31. doi: 10.2174/1381612826666201002145454, PMID: 33006535

[15] Midena E Parrozzani R Midena G Trainiti S Marchione G Cosmo E. In vivo intraocular biomarkers: changes of aqueous humor cytokines and chemokines in patients affected by uveal melanoma. Medicine (Baltimore). (2020) 99:e22091. doi: 10.1097/MD.0000000000022091, PMID: 32957329 PMC7505308

[16] Chen MX Liu YM Li Y Yang X Wei WB. Elevated VEGF-A & PLGF concentration in aqueous humor of patients with uveal melanoma following Iodine-125 plaque radiotherapy. Int J Ophthalmol. (2020) 13:599–605. doi: 10.18240/ijo.2020.04.11, PMID: 32399411 PMC7137709

[17] Usui Y Tsubota K Agawa T Ueda S Umazume K Okunuki Y. Aqueous immune mediators in malignant uveal melanomas in comparison to benign pigmented intraocular tumors. Graefes Arch Clin Exp Ophthalmol. (2017) 255:393–9. doi: 10.1007/s00417-016-3541-5, PMID: 27878431 PMC5285432

[18] Lissoni P. Therapy implications of the role of interleukin-2 in cancer. Expert Rev Clin Immunol. (2017) 13:491–8. doi: 10.1080/1744666X.2017.1245146, PMID: 27782752

[19] Teng MW Bowman EP McElwee JJ Smyth MJ Casanova JL Cooper AM. IL-12 and IL-23 cytokines: from discovery to targeted therapies for immune-mediated inflammatory diseases. Nat Med. (2015) 21:719–29. doi: 10.1038/nm.3895, PMID: 26121196

[20] Ushach I Zlotnik A. Biological role of granulocyte macrophage colony-stimulating factor (GM-CSF) and macrophage colony-stimulating factor (M-CSF) on cells of the myeloid lineage. J Leukoc Biol. (2016) 100:481–9. doi: 10.1189/jlb.3RU0316-144R, PMID: 27354413 PMC4982611

[21] Lee CS Jun IH Kim TI Byeon SH Koh HJ Lee SC. Expression of 12 cytokines in aqueous humour of uveal melanoma before and after combined Ruthenium-106 brachytherapy and transpupillary thermotherapy. Acta Ophthalmol. (2012) 90:e314–20. doi: 10.1111/j.1755-3768.2012.02392.x, PMID: 22429778

[22] Cheng Y Feng J Zhu X Liang J. Cytokines concentrations in aqueous humor of eyes with uveal melanoma. Medicine (Baltimore). (2019) 98:e14030. doi: 10.1097/MD.0000000000014030, PMID: 30702560 PMC6380850

[23] Ly LV Bronkhorst IH van Beelen E Vrolijk J Taylor AW Versluis M. Inflammatory cytokines in eyes with uveal melanoma and relation with macrophage infiltration. Invest Ophthalmol Vis Sci. (2010) 51:5445–51. doi: 10.1167/iovs.10-5526, PMID: 20538984 PMC3261048

